# Benzoxazole Derivative K313 Induces Cell Cycle Arrest, Apoptosis and Autophagy Blockage and Suppresses mTOR/p70S6K Pathway in Nalm-6 and Daudi Cells

**DOI:** 10.3390/molecules25040971

**Published:** 2020-02-21

**Authors:** Wenying Zhong, Xinwen Tang, Yang Liu, Chunyu Zhou, Pan Liu, Enhui Li, Peilin Zhong, Haoxue Lv, Qiang Zou, Maolin Wang

**Affiliations:** 1 Key Laboratory of Bio-resources and Eco-environment of the Ministry of Education, College of Life Sciences, Sichuan University, Chengdu 610065, China; hh1234qwer1@163.com; 2 Center of Science and Research, Chengdu Medical College, Chengdu 610513, China; tangxinwei11@gmail.com (X.T.); scunn519@gmail.com (Y.L.); 3 School of Pharmacy, Chengdu Medical College, Chengdu 610083, China; chunyu532@foxmail.com (C.Z.); CoooCaaa@163.com (E.L.); zhongpeiling33@163.com (P.Z.); lv1137641610@163.com (H.L.); 4 College of Biological Science and Technology, Chengdu Medical College, Chengdu 610500, China; liupan145@gmail.com

**Keywords:** K313, B-cell acute lymphoblastic leukemia, B-cell Burkitt’s lymphoma, cell cycle arrest, apoptosis, mitochondrial pathway, autophagy blockage

## Abstract

Benzoxazole derivative K313 has previously been reported to possess anti-inflammatory effects in lipopolysaccharide-induced RAW264.7 macrophages. To date, there have been no related reports on the anticancer effects of K313. In this study, we found that K313 reduced the viability of human B-cell leukemia (Nalm-6) and lymphoma (Daudi) cells in a dose-dependent manner without affecting healthy peripheral blood mononuclear cells (PBMCs) and induced moderate cell cycle arrest at the G0/G1 phase. Meanwhile, K313 mediated cell apoptosis, which was accompanied by the activation of caspase-9, caspase-3, and poly ADP-ribose polymerase (PARP). Furthermore, cells treated with K313 showed a significant decrease in mitochondrial membrane potential (MMP), which may have been caused by the caspase-8-mediated cleavage of Bid, as detected by Western blot analysis. We also found that K313 led to the downregulation of p-p70S6K protein, which plays an important role in cell survival and cell cycle progression. In addition, treatment of these cells with K313 blocked autophagic flux, as reflected in the accumulation of LC3-II and p62 protein levels in a dose- and time-dependent manner. In conclusion, K313 decreases cell viability without affecting normal healthy PBMCs, induces cell cycle arrest and apoptosis, reduces p-p70S6K protein levels, and mediates strong autophagy inhibition. Therefore, K313 and its derivatives could be developed as potential anticancer drugs or autophagy blockers in the future.

## 1. Introduction

Human B-cell acute lymphoblastic leukemia (B-ALL), which is the most common childhood cancer, is an aggressive hematological disease accounting for ~70% of acute lymphoblastic leukemia [1,2]. Although childhood B-ALL has a good treatment outcome, with a five-year survival rate of ~90%, adults usually have worse outcomes, with five-year overall survival rates of 30% to 40% [3,4,5]. Compared with a relapse rate of 20% in children, adults unfortunately have a higher relapse rate of ~50% [6,7]. Nowadays, chemotherapy is the first-line treatment of B-ALL, with chemotherapeutic agents such as vinblastine, corticosteroids, anthracycline, bleomycin, dacarbazine, doxorubicin, and gemcitabine [2,8]. An increasing number of antibody-based drugs exist, e.g., anti-CD20 antibody (rituximab, ofatumumab), anti-CD22 antibody (epratuzumab), and anti-CD25 antibody (ADCT-301) [2,9]. Furthermore, combination therapies including chemotherapy–radiotherapy and chemotherapy–biotherapy are usually used to increase survival rates for patients with relapsed or refractory acute lymphoblastic leukemia [8,10]. However, chemoresistance and adverse effects still present major limitations in the clinical treatment of B-ALL patients [1,11]. 

Human B-cell Burkitt’s lymphoma is a disease related to the Epstein–Barr virus. British surgeon Dennis Burkitt first described this kind of lymphoma, which mainly affects patients’ jaws, during his work in Africa in 1958. Chemotherapy is the main treatment method of Burkitt’s lymphoma, as it proliferates very rapidly and is sensitive to chemotherapy [12]. The principles of chemotherapy include the use of high-dose alkylating agents, high-intensity chemotherapy, central prophylaxis, and intrathecal injection of high-dose chemotherapy. Although the treatment outcome of B-cell Burkitt’s lymphoma has improved a lot in children, the prognosis is still poor in elderly adults [13,14]. Therefore, an urgency exists regarding the development of new kinds of chemotherapeutic agents with improved treatment outcomes and reduced side effects. 

Our study found that K313, a benzoxazole derivative, was effective in affecting the viability of Nalm-6 and Daudi cells. K313 is a derivative of benzoxazole, which is an aromatic organic compound that consists of a benzene-fused oxazole ring. Benzoxazole derivatives were reported to possess antiviral, antimicrobial, antibacterial, antifungal, anticancer, antidepressant, and anti-inflammatory properties [15,16,17,18,19]. Moreover, some benzoxazole derivatives, including flunoxaprofen, benoxaprofen, chlorzoxazone, and zoxazolamine, are widely used in clinical anti-inflammatory treatments [20]. We explored several mechanisms of K313 activity.

Apoptosis plays an important role in the development and homeostasis of many diseases. It contains three different pathways, including the death-receptor-mediated pathway, the endoplasmic reticulum stress pathway, and the mitochondrial pathway, involving many Bcl-2 family members (bax, bad, Bid, and Bcl-2), caspase-8, -9, -7, -6, -3, and PARP. The mitochondrial apoptosis pathway is associated with the regulation of related proteins, the disruption of mitochondrial transmembrane potential, the production of superoxide radicals, the release of apoptosis-inducing factor (AIF), and so on. Caspase-8, as a member of the death-inducing signaling complex (DISC), is a key factor in transmitting apoptosis signals of the Fas/FasL pathway. Activated caspase-8 can cut 22 kDa Bid in the cytoplasm into a 15 kDa protein termed truncated Bid (t-Bid), which is then translocated into the mitochondria and influences mitochondrial outer-membrane permeability, resulting in amplification of apoptosis signals [21]. 

Autophagy, meaning “self-eating” was first found in yeast and involves the degradation of long-lived, misfolded proteins and dysfunctional or damaged organelles [22,23]. There are three general types of autophagy, including microautophagy, chaperone-mediated autophagy, and macroautophagy, which we often call “autophagy” [24]. Autophagy is induced by nutrient deprivation, hypoxia, stress, and some chemical drugs such as rapamycin, a well-known autophagy inducer targeting mTOR (mammalian target of rapamycin) [25,26]. In normal cells, autophagy plays an important role in regulating the homeostatic balance, whereas in cancer cells, autophagy is a “double-edged sword” [27,28]. Early reports documented that autophagy was initially believed to be a tumor-suppression mechanism. BECLIN1/ATG6, a key autophagy-related gene, was monoallelically lost in more than 40% of human prostate, breast, and ovarian cancers [29,30]. In contrast to Becn1+/+ mice, Becn1+/− mice had a higher risk of lung cancer, hepatocellular carcinoma, and lymphoma [31,32]. Conversely, accumulating evidence reveals that autophagy may play a role in promoting cancer development, therefore making it a potential anticancer target as previously reviewed [33]. Degradation of autophagic cargo provides amino acids, fatty acids, glucose, and nucleotides for cancer cells, which then promotes the survival and growth of cancer cells. When tumor tissues are deprived of oxygen, autophagy is upregulated, thus providing rescue nutrients and energy for cellular metabolism in anoxic regions [34,35]. Cancer cells have a relatively high levels of autophagy tend to adapt to a vigorous metabolism when compared with normal cells [36]. Some cancer cells undergo autophagy-mediated cell death by anticancer therapies, such as chemotherapy, radiotherapy, and target therapy [36], whereas some cancer cells may go through autophagy-mediated protective function and mediate drug resistance to these treatments [37,38]. Various studies demonstrated that inhibition of autophagy enhanced the toxicity of anticancer agents and decreased chemoresistance in osteosarcoma, ovarian cancer stem cells, melanoma, lymphoma, endometrial cancer and hepatocellular carcinoma [38,39,40,41]. Therefore, in some cancer cells, autophagy inhibition exerts anticancer effects; for example, chloroquine (CQ) and hydroxychloroquine (HCQ), which are inhibitors of autophagy that change lysosome acidification and inhibit autophagosome degradation by lysosomes, was combined with radiation and carmustine to treat glioblastoma in a phase III clinical trial [25]. 

In the present study, we firstly observed that K313 markedly induced autophagy blockage, apoptosis, and cell cycle arrest in Nalm-6 and Daudi cells. In the future, K313 and its derivatives may be developed as new chemotherapy or autophagy-blocking agents.

## 2. Results

### 2.1. K313 Reduces the Viability of Nalm-6 and Daudi Cells without Affecting Normal Healthy PBMCs

**Figure 1:**
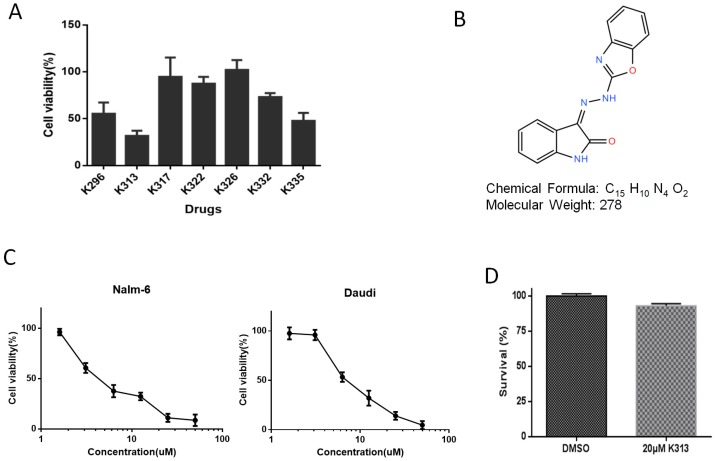
K313 reduces the viability of Nalm-6 and Daudi cells in vitro. (**A**) The cytotoxicity of several drugs were simply tested in a single concentration (10 µM) by treating Nalm-6 cells for 48 h. (**B**) Chemical structure of K313. (**C**) Nalm-6 and Daudi cells were treated with different concentrations (1.6, 3.1, 6.3, 12.5, 25.0, 50.0 µM) of K313 for 48 h. The viability of the Nalm-6 and Daudi cells decreased in a dose-dependent manner. (**D**) Survival of PBMCs after incubation with 20 µM K313 for 48 h. Cells were stained with 7-aminoactinomycine-D (7-AAD) and analyzed using a flow cytometer. 0.5% dimethyl sulfoxide (DMSO) treated control cells represent 100% survival.

The cytotoxicity levels of several drugs were tested using a single concentration (10 µM) by treating Nalm-6 cells for 48 h. Among them, K313 was found to be relatively the most effective (Figure 1A). Next, Nalm-6 and Daudi cells were treated with different concentrations of K313 for 48 h. Cell Counting Kit-8 (CCK-8) was used to evaluate cell viability after treatment of different concentrations of K313. We found that K313 markedly decreased the viability of Nalm-6 and Daudi cells in a dose-dependent manner (Figure 1C). The half inhibition concentration (IC50) values were calculated using Graphpad prism 6 software, with the IC50 values of Nalm-6 and Daudi cells being 3.4 µM and 6.4 µM, respectively. In contrast, we found that K313 almost had no cytotoxicity to normal, healthy PBMCs even with a concentration of 20 µM (Figure 1D), implying that K313 has less toxicity and few potential side effects to normal healthy cells. 

### 2.2. K313 Induces Moderate Cell Cycle Arrest at G0/G1 Phase in Nalm-6 and Daudi Cells

**Figure 2:**
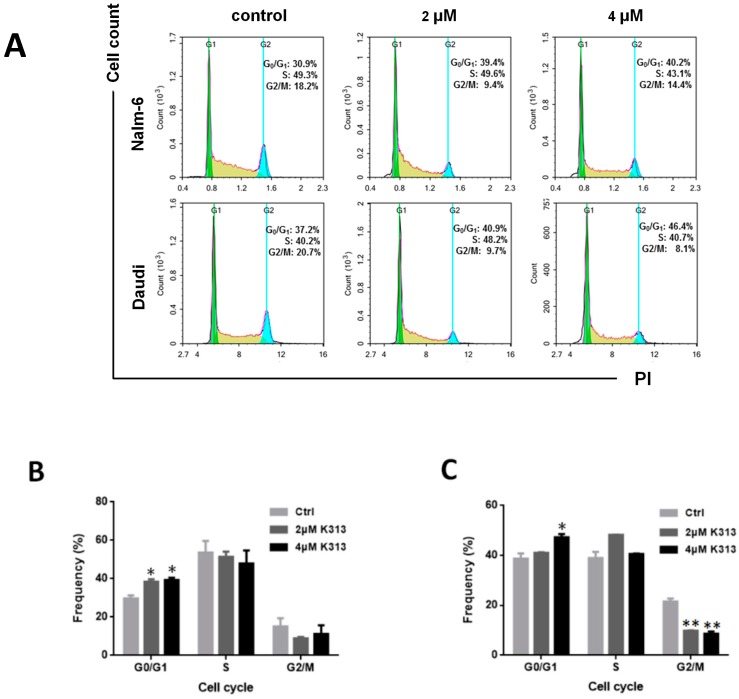
K313 induces Nalm-6 and Daudi cell cycle arrest moderately at the G0/G1 phase. (**A**) Nalm-6 and Daudi cells were treated with different concentrations of K313 for 24 h. The cells were washed, fixed, and stained with PI and analyzed for DNA distribution by flow cytometry. The percentages of (**B**) Nalm-6 cells and (**C**) Daudi cells in different phases of the cell cycle are shown. The corresponding percentage values of Nalm-6 and Daudi cells in the G0/G1, S, and G2–M phase were calculated. * *p* < 0.05 and ** *p* < 0.01 vs. control (0.1% DMSO) group.

To further investigate whether the cell viability reduction effect of K313 was related to cell cycle arrest, we analyzed the cell cycle distribution by propidium iodide (PI) staining and flow cytometry. We found that the percentage of G0/G1 phase distribution of K313-treated cells increased compared with control. As shown in Figure 2, 4 µM K313 increased G0/G1 phase distribution induction from 30.9% to 40.2% in Nalm-6 cells and from 37.2% to 46.4% in Daudi cells. These results demonstrated that K313 arrested Nalm-6 and Daudi cell cycle at the G0/G1 phase, which may have contributed to the cell viability reduction effect of K313.

### 2.3. K313 Induces Apoptosis in Nalm-6 and Daudi Cells

**Figure 3:**
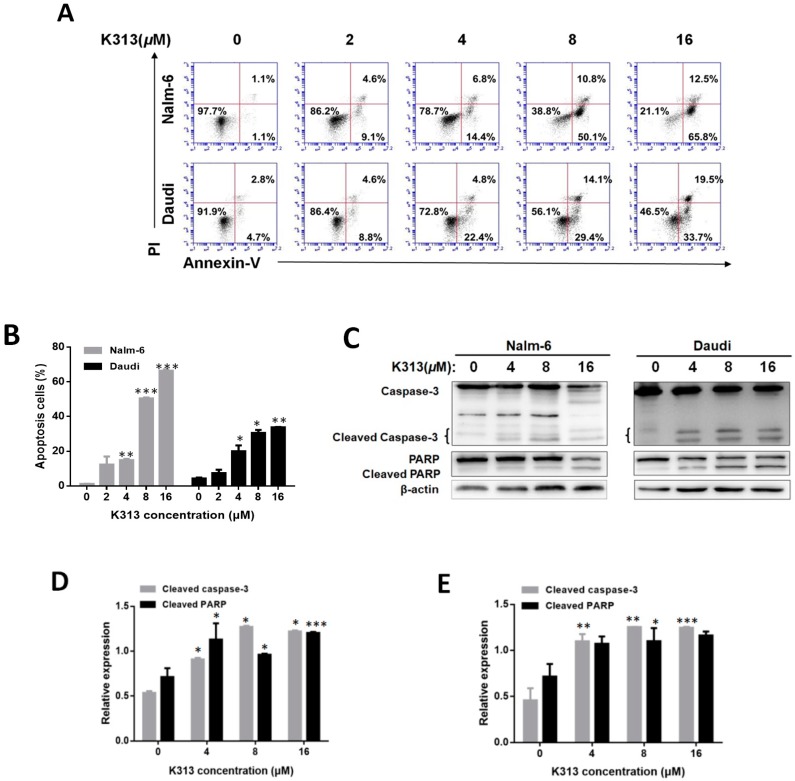

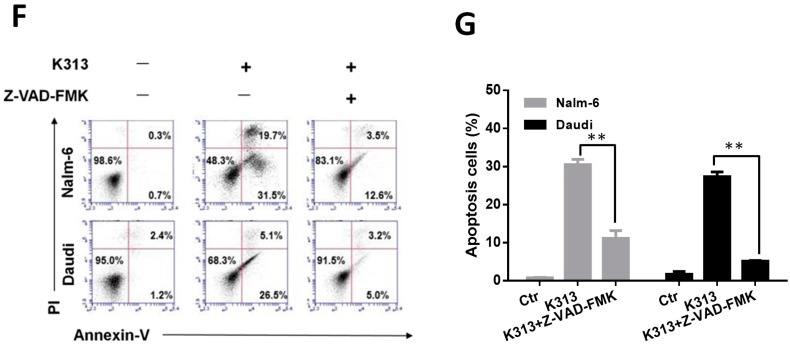
K313 induces apoptosis in Nalm-6 and Daudi cells. (**A**) Nalm-6 and Daudi cells were incubated with varying concentrations of K313 for 48 h. Cells were harvested and incubated with Annexin V-FITC and PI and then analyzed using flow cytometry (FCM). (**B**) The percentage of apoptotic cells was evaluated in Nalm-6 and Daudi cells. (**C**) Nalm-6 and Daudi cells were treated with K313 (0, 4, 8, and 16 µM) for 48 h. The cells were harvested and the whole protein lysates were subjected to Western blot analysis. The apoptotic protein expression levels in (**D**) Nalm-6 and (**E**) Daudi cells were quantified by Quantity One software. (**F**) Nalm-6 and Daudi cells were treated with 20 µM K313 only or a combination of 20 µM K313 and 50 µM Z-VAD-FMK (an irreversible pan-caspase inhibitor), and the cells were harvested and incubated with Annexin V-FITC and PI and analyzed by FCM. (**G**) The percentage of apoptotic cells was quantified in the control (0.2% DMSO), K313 only, and combination of K313 and Z-VAD-FMK. * *p* < 0.05, ** *p* < 0.01, and *** *p* < 0.001 vs. control group.

In addition to cell cycle arrest function, apoptosis may still play an important role in the cell viability reduction effect of K313. Therefore, Nalm-6 and Daudi cells were incubated with different concentrations of K313 for 48 h. Then, after Annexin V-FITC (fluorescein isothiocyanate) and PI fluorescence staining, the percentage of apoptosis-positive cells was measured by flow cytometry. As shown in Figure 3A, K313 induced cell apoptosis in a dose-dependent manner. In Nalm-6 cells, 2 µM and 16 µM K313 treatments for 48 h induced cell apoptosis-positive rates of 9.1% and 65.8%, respectively. In Daudi cells, 16 µM K313 increased apoptosis rate induction from 4.7% to 33.7% compared to the control. According to these results, in terms of apoptosis induction ability of K313, Nalm-6 cells were more sensitive to K313 than Daudi cells (Figure 3B). Less apoptosis induction effects were observed when the cells were treated with K313 for 24 h (Figure S1). Next, the expression levels of apoptosis-associated proteins (caspase-3, PARP) were examined by Western blotting. K313 activated caspase-3 and PARP, resulting in these proteins being cleaved into small active fragments in both cell lines (Figure 3C–E). To further investigate whether K313 induced apoptosis was specifically associated with caspase activation, we explored whether Z-VAD-FMK affected apoptosis for 12 h as a classic caspase inhibitor. As shown in Figure 3F,G, compared with the K313-only group, the percentage of apoptotic cells greatly decreased in Nalm-6 and Daudi cells in the combination group of K313 and Z-VAD-FMK. These results demonstrated that K313 induced apoptosis in Nalm-6 and Daudi cells and may play an important role in the cell viability reduction effect of K313.

### 2.4. K313 Decreases Cell Mitochondrial Membrane Potential and Activates Mitochondrial Pathway of Apoptosis

**Figure 4:**
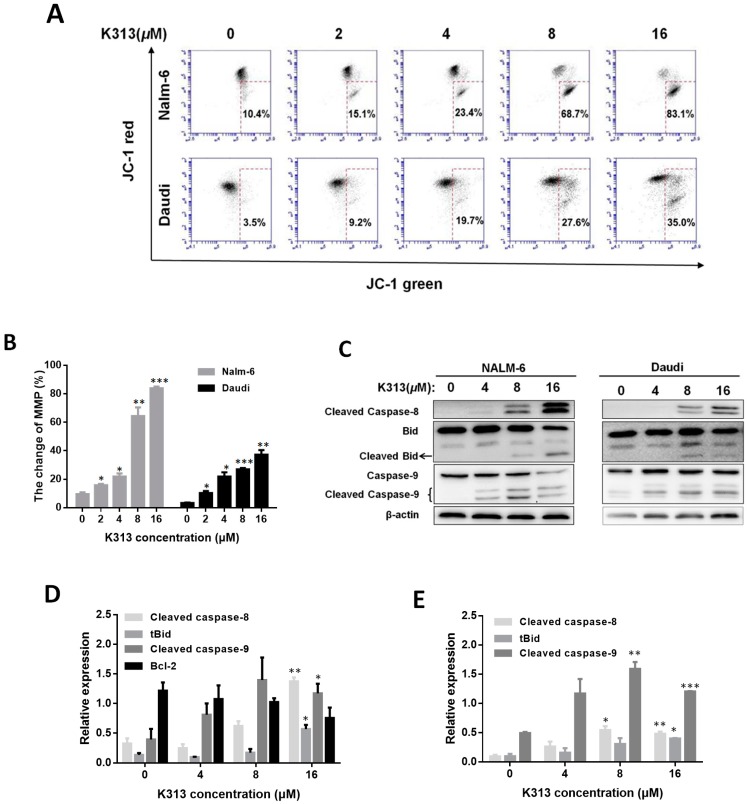
K313 induces apoptosis in Nalm-6 and Daudi cells through the mitochondrial pathway. (**A**) Nalm-6 and Daudi cells were treated with K313 at indicated concentrations for 48 h and stained with JC-1 for 15 min. Loss of mitochondrial membrane potential was measured by FCM. (**B**) Columns represent the percentage of change of the MMP in Nalm-6 and Daudi cells. (**C**) After treatment with the vehicle or K313 for 24 h, the total protein lysates were detected by immunoblotting with antibodies. The expression levels of apotosis-related proteins in (**D**) Nalm-6 and (**E**) Daudi cells were quantified by Quantity One software. * *p* < 0.05, ** *p* < 0.01, and *** *p* < 0.001 vs. control group.

In order to further investigate the mechanism of apoptosis in K313-treated Nalm-6 and Daudi cells, the mitochondrial membrane potential (MMP) was examined and the mitochondrial pathway-related proteins were analyzed. It is well known that cell mitochondria participate in the regulation of apoptosis and decreases in MMP coincide with membrane permeability and mitochondrial dysfunction [42]. JC-1 staining was used to detect the MMP in this study. Normal cells usually have high MMP, enabling them to form JC-1 aggregates and showing red fluorescence. When MMP decreases, JC-1 exists in its monomeric form and shows green fluorescence. Therefore, there is a shift from red JC-1 aggregates to green JC-1 monomers when MMP decreases [43]. K313 treatment for 48 h apparently depolarized the MMP in a dose-dependent manner in both the Nalm-6 and Daudi cells (Figure 4A). In contrast, K313 treatment for 24 h showed less effects on MMP (Figure S2). Next, Western blot analysis was used to determine the level of mitochondrial pathway-related proteins, such as Bid, Bcl-2, caspase-8, and caspase-9. After treatment with K313, activation of caspase-8, caspase-9, and Bid was found in both the Nalm-6 and Daudi cells, but the protein level of Bcl-2 did not change in the Nalm-6 cells. It is worth noting that the Daudi cells do not express Bcl-2 protein, as reported previously (Figure 4C) [44]. Collectively, we found that K313 decreased cell MMP and activated the mitochondrial pathway of apoptosis.

### 2.5. K313 Suppresses mTOR/p70S6K Pathway in Nalm-6 and Daudi Cells

**Figure 5:**
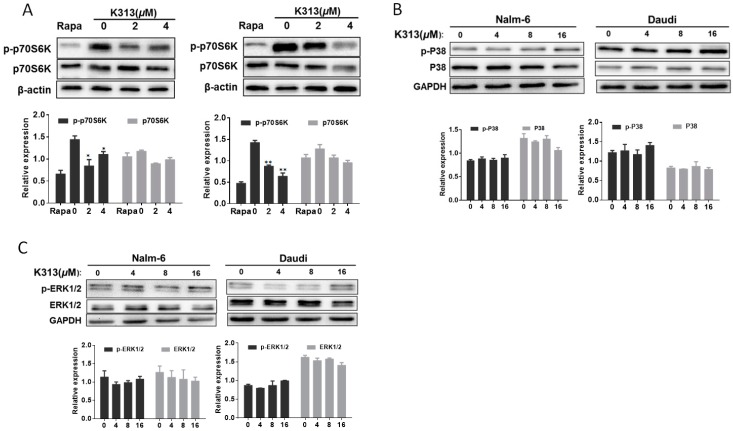
K313 downregulates the phosphorylation of p70S6K in Nalm-6 and Daudi cells. Nalm-6 and Daudi cells were treated with K313 at the indicated concentrations for 12 h. (**A**) Protein levels of p-p70S6K were determined by Western blotting after treatment with K313 or rapamycin. Related protein expression levels were quantified by Quantity One software (Bio-Rad). (**B**,**C**) Protein levels of p-P38 and p-ERK1/2 were analyzed by Western blotting. Related protein expression levels were quantified by Quantity One software (Bio-Rad). * *p* < 0.05 and ** *p* < 0.01 vs. control group.

P70S6K, the downstream molecule of mTOR (the mammalian target of rapamycin), participates in ribosomal biogenesis and the selective translation of special mRNA populations [45]. The well-known mTOR/p70S6K signaling pathway is activated in many human cancers and plays an important role in cell survival and cell cycle progression [46,47,48]. Here, we found that rapamycin targeting mTOR greatly downregulated p-p70S6K in Nalm-6 and Daudi cells. Treatment with low-concentration K313 also obviously suppressed the phosphorylation of p70S6K in Nalm-6 and Daudi cells, thereby revealing a potent mechanism of cell cycle arrest from K313 (Figure 5A). In order to explore whether K313 affected the MAPK pathway, we examined the protein expression levels of p-ERK and p-P38 of K313-treated cells. K313 did not affect the phosphorylation of ERK and P38 in Nalm-6 and Daudi cells after 12 h of treatment. (Figure 5B,C).

### 2.6. K313 Inhibits Autophagic Flux in Cancer Cells

**Figure 6:**
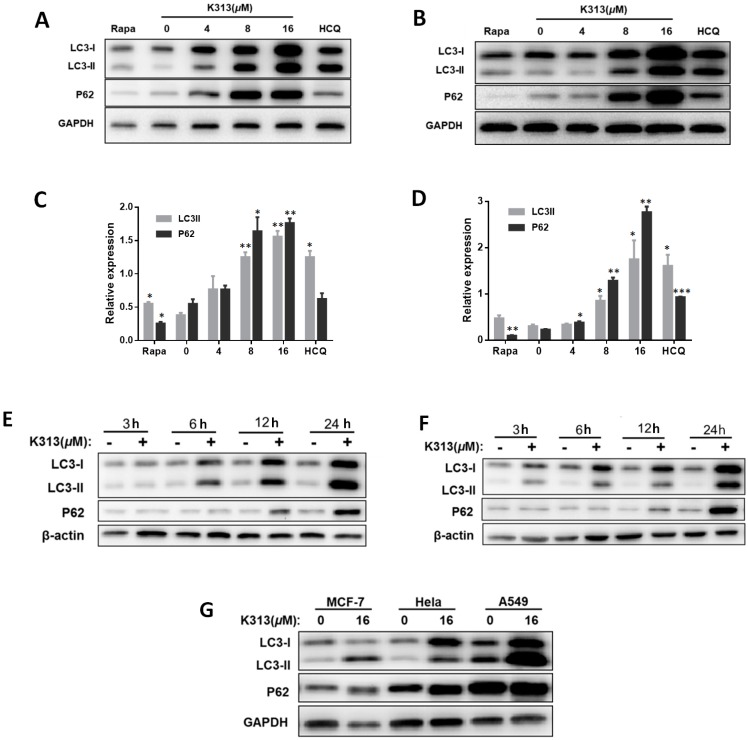
K313 induces autophagy blockage in Nalm-6 and Daudi cells in a dose- and time-dependent manner. Different concentrations of K313, 5 μM rapamycin, and 20 μM hydroxychloroquine (HCQ) were used to treat (**A**) Nalm-6 and (**B**) Daudi cells for 24 h. Then, the whole cell lysates were analyzed by Western blotting using LC3 and P62 antibodies. Quantitative analyses of protein levels were conducted in (**C**) Nalm-6 and (**D**) Daudi cells. Further, 8 µM K313 was used to treat (**E**) Nalm-6 and (**F**) Daudi cells at different time-points, and the total cell lysates were analyzed by Western blot. (**G**) After incubation with or without 16 µM K313 for 24 h in MCF-7, Hela, and A549 cells, the whole protein lysates were tested by Western blot using LC3 and P62 protein antibodies. * *p* < 0.05, ** *p* < 0.01, and *** *p* < 0.001 vs. control group.

To investigate the effects of K313 on autophagy in vitro, we tested several types of cancer cells. Microtubule-associated protein light chain 3 (LC3) is a well-established marker for autophagy activation. At the early stage of autophagy, cytoplasm-localized LC3-I is lipidated to generate LC3-II, which is recruited to autophagosomal membranes. When autophagosomes fuse with lysosomes to form autolysosomes, LC3-II is degraded by lysosomal hydrolases [49]. Therefore, the conversion of LC3-I to LC3-II or the degradation of LC3-II are hallmarks of autophagic flux monitoring, but the latter is more appropriate [50]. In addition, p62 is regarded as a marker for monitoring autophagic flux. At the late stage of autophagy, p62-bound polyubiquitinated proteins are incorporated into the completed autophagosome and are degraded in autolysosomes. Thus, the accumulation of LC3-II-labeled autophagosomes or p62 aggregates are robust signs of autophagic flux inhibition [51]. Our study showed that rapamycin, known as a classic autophagy inducer, promoted the degradation of the p62 protein and the conversion of LC3-I to LC3-II in Nalm-6 and Daudi cells [52]. Interestingly, we found that K313 treatment resulted in accumulation of p62 and LC3-II proteins, similar to HCQ (a well-known autophagy inhibitor), indicating that K313 could induce dose-dependent autophagic blockage (Figure 6A,B). In order to test whether K313-mediated autophagic blockage was time-dependent, we measured the protein levels of LC3-II and p62 at different time points. The results optimistically showed that K313 caused a substantial accumulation of LC3-II and p62 in a time-dependent manner (Figure 6E,F). Next, we tested whether K313 also induced autophagy in other types of cancer cells, including MCF-7, Hela, and A549; we found that both p62 and LC3-II proteins accumulated after K313 treatment (Figure 6G). Furthermore, by using immunofluorescence staining, increased endogenous LC3 puncta was observed in the presence of K313 or HCQ in Hela cells (Figure S3). These results implied that K313 induces autophagy blockage, suggesting that K313 may serve as a potential autophagic flux inhibitor.

## 3. Discussion

Currently, chemoresistance is still one of the major obstacles preventing successful B-cell leukemia and lymphoma treatment. Clinical chemotherapy such as bortezomib, adriamycin, and vinblastine are used to treat relapsed or refractory patients, but the outcomes are still not satisfactory [53]. In recent years, some novel cellular-based anticancer immunotherapies rapidly appeared. Among them, anti-CD19/CD22 chimeric antigen receptor T-cell (CAR-T) therapy, which targets relapsed or refractory patients, has been extremely efficient and promising. Although CAR-T is an attractive option to treat leukemia or lymphoma, it sometimes needs to be combined with other chemotherapeutic agents to get the best outcome [54]. Overall, new kinds of chemotherapeutic agents are still largely required by the scientific community [1,8].

**Scheme 1:**
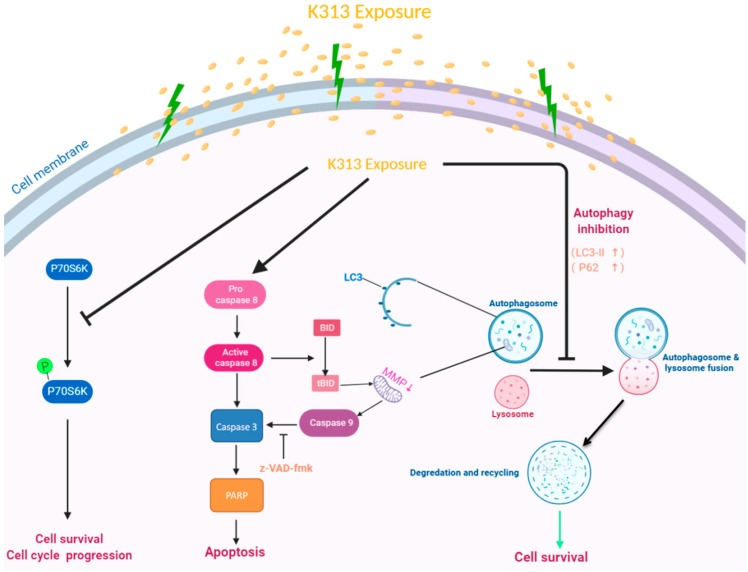
The proposed mechanisms of K313-mediated cell death. Exposure of cells to K313 affects many aspects, such as the reduced phosphorylation of p70S6K, the regulation of apoptosis-related proteins, and the inhibition of autophagy, marked by the accumulation of LC3-II and P62 proteins.

A previous study showed that the benzoxazole derivative K313 demonstrated anti-inflammatory effects by affecting the secretion of pro-inflammatory cytokines, such as nitric oxide (NO), TNF-α, and IL-6 via GSK-3β inhibition in LPS-induced RAW264.7 macrophages. Further, they observed no significant change in the protein levels of p38 MAPK and ERK1/2, which was confirmed again in our study [55]. Until now, the anticancer effects of K313 have not been explored. Here, we firstly demonstrated the anticancer effects of K313 and further explored the related mechanisms. Based on our investigations, we propose several mechanisms of K313-mediated cell death (Scheme 1). K313 reduced the viability of Nalm-6 and Daudi cells in a dose-dependent manner (Figure 1B). Notably, the toxicity of K313 to PBMCs was negligible, implying that K313 has much less toxicity in normal healthy cells than cancer cells. The cell cycle was then analyzed by FCM, which showed that K313 moderately inhibited the cell cycle at the G0/G1 phase, thereby potentially contributing to the cell viability reduction effect of K313. Next, the apoptosis-induction function of K313 was confirmed and we further tried to explore the underlying mechanism of apoptosis.

The mitochondrial membrane potential (MMP) is related to mitochondrial membrane permeability, which changes according to H^+^/K^+^-ATPases, proton pumps, or some mitochondrial membrane proteins, such as t-Bid, Bcl-2, bax, and so on. Earlier research indicated that Bid could be cleaved into t-Bid by activated caspase-8 in the cytosol, allowing t-Bid to translocate to the mitochondria and alter mitochondrial outer-membrane permeabilization [56]. Our experiments found that K313 strongly changed the mitochondrial membrane potential in a dose-dependent manner in Nalm-6 and Daudi cells (Figure 4A). Meanwhile, K313 increased the expressive level of cleaved-Bid, -8, -3, -9 and -PARP, but did not affect Bcl-2 activation (Figure 4C). These data together indicated that cleavage of Bid by caspase-8 might mediate mitochondrial damage and that K313 induced cell apoptosis through the mitochondrial pathway. K313 treatment also downregulated p-p70S6K protein levels, which may have caused cell cycle arrest at the G0/G1 phase and cell survival disruption, as previously reported by others [46].

Many reports indicate that autophagy inhibition exerts anticancer effects [57,58,59]. A previous study showed that bortezomib-mediated autophagy inhibition promoted this anticancer activity against Nalm-6 cells [60]. Similarly, in our study, we observed the strong autophagy blockage effect of K313 in Nalm-6 cells. As known, autophagy clears damaged and senescent mitochondria [61]. We speculate that in K313-treated cells, autophagy inhibition might lead to the accumulation of dysfunctional mitochondria, resulting in disrupted energy metabolism and triggering apoptosis. Furthermore, we found that p62 and LC3-II proteins also accumulated in other types of cancer cells after K313 treatment (Figure 6A,B), suggesting that K313 acts as a potent inhibitor of autophagy in various cancer cell types, which may be utilized by the scientific community in cancer treatment in the future. 

Taken together, our study demonstrated that K313 induces apoptosis via a mitochondrial signaling pathway, mediates strong autophagy blockage, and downregulates the phosphorylation of p70S6K, which may cause cell cycle arrest at the G0/G1 phase in Nalm-6 and Daudi cells. Therefore K313 could be developed as a potential lead chemical compound in anticancer drug discovery. Further investigations should include uncovering more detailed mechanisms of K313, broadening the anticancer scope of K313, and testing the combination effects of K313 and other treatment strategies (including cell-based immunotherapy, which is currently ongoing in our laboratory).

## 4. Materials and Methods

### 4.1. Cells and Reagents

Benzoxazole derivative K313 (Compound ID: 5939009) was obtained from ChemBridge Corporation (San Diego, CA, USA), which was dissolved in DMSO at 10 mM as stock solution. Normal, healthy peripheral blood mononuclear cells (PBMCs) were isolated from human whole blood with Ficoll-Paque. Human B-cell leukemia (Nalm-6) and lymphoma (Daudi) cell lines were purchased from Feiouer Bio-Technique Co., Ltd. (Chengdu, China). Other types of cancer cell lines, including MCF-7 (breast), Hela (cervix), and A549 (lung) cell lines, were kindly provided by Dr. Tai Yang from Chengdu Medical College. All the cell lines were characterized by Feiouer using short tandem repeat (STR) markers; the STR authentication reports are provided in the supplementary materials. Cell Counting Kit-8 (CCK-8), a cell viability detection reagent, was purchased from Dojindo (Kumamoto, Japan). Annexin V-FITC/PI Apoptosis Detection Kit was purchased from KeyGEN BioTECH. JC-1 (5,5′,6,6′-tetrachloro-1,1′,3,3′-tetraethylbenzimidazolylcarbocyanine iodide) dye reagent used to detect MMP was supplied by MedChemExpress (Monmouth Junction, NJ, USA). The first antibodies of caspase-9, caspase-8, caspase-3, PARP, Bid, Bcl-2, ERK1/2, p38, p70S6K, p-ERK1/2, p-P38, p-p70S6K beta-actin, and GAPDH were purchased from Cell Signaling Technology (Beverly, MA, USA). Anti-LC3 antibody was purchased from Novus Biologicals (Littleton, CO, USA). Anti-p62 antibody was obtained from Boster Biological Technology (Wuhan, China). Enhanced chemiluminescence reagent was obtained from Millipore (Bedford, MA, USA). All other chemicals used in our experiments were of analytical grade.

### 4.2. Cell Culture

Nalm-6 and Daudi cell lines were maintained at the proper density in Roswell Park Memorial Institute 1640 medium supplemented with 10% fetal bovine serum (FBS) (Millpore). The Hela, MCF-7 and A549 cell lines were cultured in Dulbecco’s modified Eagle’s medium (DMEM) supplemented with 10% FBS. Normal, healthy peripheral blood mononuclear cells (PBMCs) were isolated from human whole blood with Ficoll-Paque and then cultured in RPMI-1640 medium supplemented with 10% FBS. All cell lines were kept in air with 5% CO2 at 37 °C.

### 4.3. Cell Viability Assay

The CCK-8 Kit was used to assay the toxicity of K313 to cancer cells. Nalm-6 or Daudi cells were seeded in flat bottom 96-well microtiter plates at a density of 5 × 10^4^ per well and then cultivated overnight. These cells were exposed to various concentrations of K313 in 96-well plates for 48 h, then 10 µL of CCK-8 detection reagent was added to each well and the plate was incubated at 37 °C for 4 h. The absorbance of each well at a wavelength of 450 nm was measured using a Spectra microplate spectrophotometer (BioTek Instruments, Winooski, VT, USA). The median inhibitory concentration (IC50) of the drug was calculated with GraphPad Prism 6 software (GraphPad Software Inc., San Diego, CA, USA). In addition, to test whether K313 would affect the survival of normal healthy cells, we treated PBMCs with 20 µM K313 for 48 h. Cells were then stained with 7-ADD and analyzed using a flow cytometer. DMSO (0.2%)-treated control cells represented 100% survival.

### 4.4. Cell Cycle Analysis

Nalm-6 and Daudi cells were cultured at a density of 1 × 10^6^ cells/mL with varying concentrations of K313 (0, 2, 4 µM) for 24 h. The cells were then harvested and washed with ice-cold phosphate-buffered saline (PBS) and fixed overnight with 70% (*v*/*v*) ethanol at 4 °C. The next day, the cells were pretreated with RNAse for 30 min at 37 °C after being washed with ice-cold PBS. Later, propidium iodide (PI) was used to stain the DNA of these cells for 30 min at 4 °C in the dark. The cell cycle profiles were measured by flow cytometry as quickly as possible. Cell cycle distribution was analyzed by NovoExpress software (Version 1.0, ACEA Biosciences Inc., San Diego, CA, USA). 

### 4.5. Detection of Cell Apoptosis

Cells were seeded at a density of 8 × 10^5^ cells per well in a 12-well plate. After treatment with K313 at different concentrations (0, 2, 4, 8, 16, 20 µM) or 50 uM Z-VAD-FMK for different time periods as indicated respectively, cells were harvested, washed, and stained using Annexin V-FITC/PI Apoptosis Detection Kit (Beckman Coulter, Fullerton, CA, USA), according to the manufacturer’s instructions. Samples were analyzed using the BD (Business Development) Accuri C6 flow cytometer (BD Accuri, San Jose, CA, USA).

### 4.6. Measurement of Mitochondrial Membrane Potential

Cells were treated with different concentrations of K313 (0, 2, 4, 8, 16 µM). After incubation, cells were collected and stained with 5 µM JC-1 fluorescent dye probe for 15 min at 37 °C. The change in mitochondrial membrane potential was then measured using the BD Accuri C6 flow cytometer, and the fluorescence emission shift from green (~529 nm) to red (~590 nm) was measured using flow software.

### 4.7. Western Blot Analysis

Cells were seeded at a density of 5 × 10^5^ cells in each well of a 12-well plate. After treatment with various concentrations of K313, Nalm-6 and Daudi cells were harvested and lysed using sodium dodecyl sulfate (SDS) loading buffer on ice. Samples were separated using SDS-PAGE electrophoresis and the separated proteins were blotted onto polyvinylidenefluoride (PVDF) membranes (Roche Company, Basel, Switzerland). Then, the hybrid membranes were blocked for 1 h with 5% nonfat milk in TBST buffer (Tris-Buffered Saline containing 0.05% Tween 20) and incubated with the related primary antibodies diluted with primary antibody dilution buffer (Beyotime Institute of Biotechnology, Shanghai, China) overnight at 4 °C. After washing at least three times, horseradish peroxidase (HRP)-conjugated secondary antibodies, anti-rabbit or anti-mouse IgG-HRP (Cell Signaling Technology, Danver, MA, USA), were added and incubated for 2 h at room temperature. After washing, the membranes were incubated with enhanced chemiluminescence reagent (ECL) and exposed to X-ray film. The immunoreactive bands were quantified using Quantity One software (Bio-Rad, Hercules, CA, USA).

### 4.8. Cell Immunofluorescence Staining

In this study, immunofluorescence staining was performed to detect autophagosomes or autolysosomes. Hela cells were treated with K313, HCQ, and rapamycin for 24 h and fixed with 4% paraformaldehyde in PBS for 15 min at room temperature, then washed 3 times and permeated for 30 min using 10% goat serum containing 0.3% Triton X-100 in PBS. Then, the cells were washed and incubated with anti-LC3 antibody (1:500 dilution, NOVUS) for 2 h and Alexa Fluor 488-conjugated goat anti-rabbit IgG (1:400 dilution, Invitrogen, Carlsbad, CA, USA) for 1 h at room temperature. Immunofluorescence observation was performed by fluorescence microscopy.

### 4.9. Statistical Analysis

The data were presented as the means ± standard deviation (SD) and compared with Student’s t-test by GraphPad Prism 6 (GraphPad software Inc., San Diego, CA, USA). A *p*-value of < 0.05 was considered statistically significant. 

## Supplementary Materials

The following are available online: Figure S1: Detection of apoptosis in Nalm-6 and Daudi cells after treated with K313 for 24 h; Figure S2: Detection of the loss of the mitochondrial membrane potential in Nalm-6 and Daudi cells after treatment with K313 for 24 h; Figure S3: Observation of LC3 fluorescent puncta in Hela cells under different treatments.
