# Supplementary material for: Benzoxazole Derivative K313 Induces Cell Cycle Arrest, Apoptosis and Autophagy Blockage and Suppresses mTOR/p70S6K Pathway in Nalm-6 and Daudi Cells
Source: Molecules. 2020 Feb 21;25(4):971. doi: 10.3390/molecules25040971 (PMC7070478; doi:10.3390/molecules25040971)
Supplement: Supplementary file 1 [file molecules-25-00971-s001.zip › Supplementary Materials/STR Authentication A-549.pdf]

Report of Human Cell Line Authentication

Analysis Date: 2017/11/01

Methods and Procedures

1. DNA is amplified with STR Multi-amplification Kit (Fluorescence Detection Kit);
2. PCR products are assayed with DNA Analyzer (Applied Biosystems®);
3. Comparision of the sample STR loci with an International Database is shown.

Results

1. The STR profiles of the sample are shown in Figure 1.
2. The comparision information is shown in Figure 2.
3. Conclusion ① is made according to Authentication of Human Cell Lines by STR DNA Profiling Analysis.
4. Conclusion ② is made according to an article from Nature Magazine.

Conclusions

① **Obvious contamination of other human cell line is not found (Figure 1).**

② **According to the comparison information, the sample matches with “A549”.**

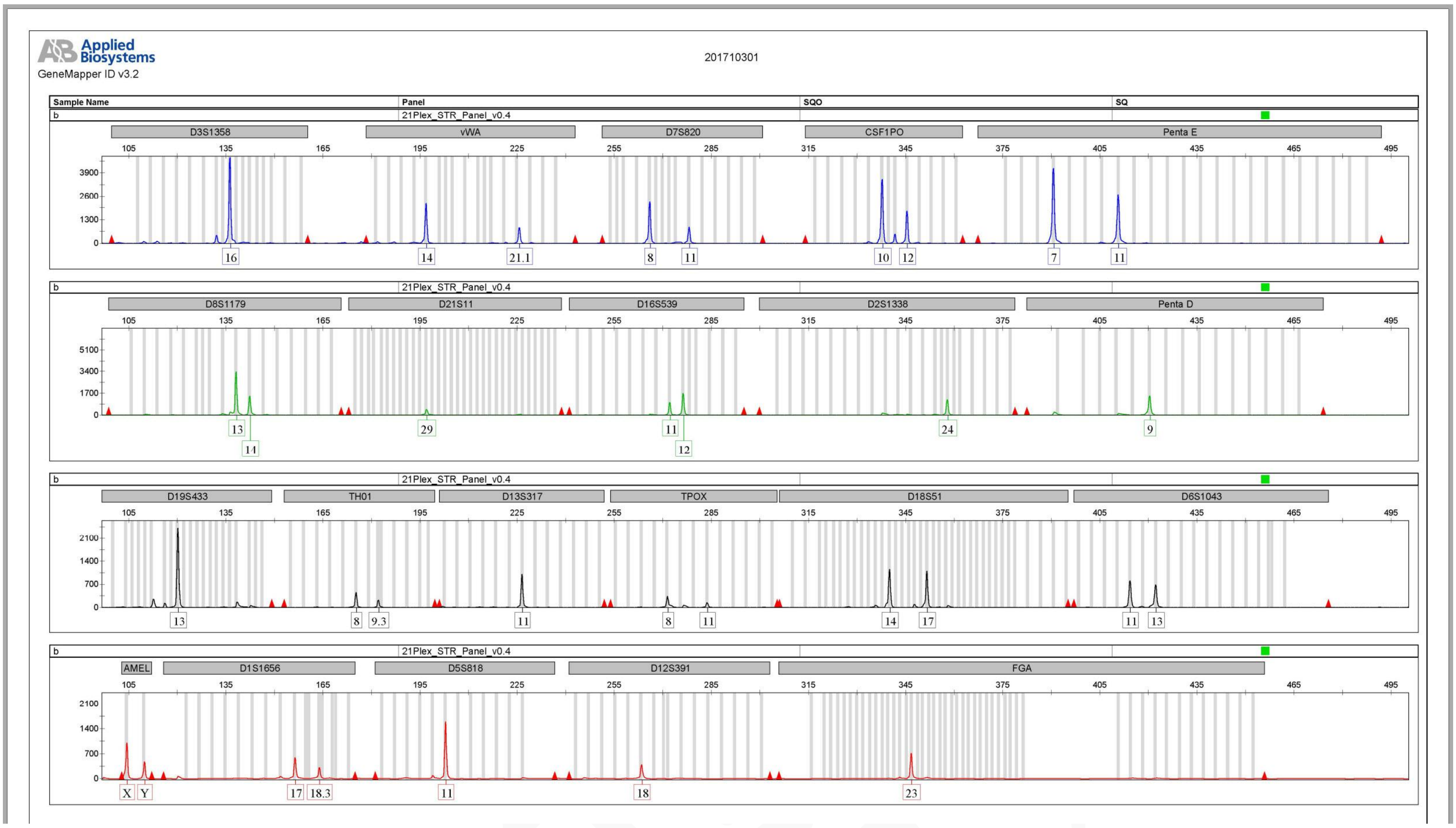

Figure 1. STR profiles of the sample

Result of STR matching analysis by your data.

- DSMZ Profile Database -

A graphical presentation is shown at the bottom of this page.

EV	Cell No.	Cell name	D5S818	D13S317	D7S820	D16S539	VWA	TH01	AM	TPOX	CSF1PO	Figures
	Query (Your Cell)		11, 11	11, 11	8, 11	11, 12	14, 21. 1	8, 9. 3	x, y	8, 11	10, 12	
0.94(34/36)	107	A-549	11, 11	11, 11	8, 11	11, 12	14, 14	8, 9. 3	X, Y	8, 11	10, 12	-

Figure 2. The comparision of the sample STR loci with an International Database

References:

[1] M. Yu, S. K. Selvaraj, M. M. Liang-Chu, S. Aghajani, M. Busse, J. Yuan, G. Lee, F. Peale, C. Klijn, R. Bourgon, J. S. Kaminker, and R. M. Neve, 'A Resource for Cell Line Authentication, Annotation and Quality Control', Nature, 520 (2015), 307-11.

[2] Yvonne Reid, PhD,1 Douglas Storts, PhD,2 Terry Riss, PhD,3,* and Lisa Minor,PhD4,*Authentication of Human Cell Lines by Str DNA Profiling Analysis', (2013).

[3] John Butler, 'Short Tandem Repeat Typing Technologies Used in Human Identity Testing', BioTechniques, 43 (2007), Sii-Sv.

[4] Hao Fan, and Jia-You Chu, 'A Brief Review of Short Tandem Repeat Mutation', Genomics, Proteomics & Bioinformatics, 5 (2007), 7-14.

[5] L. P. Freedman, M. C. Gibson, S. P. Ethier, H. R. Soule, R. M. Neve, and Y. A. Reid, 'Reproducibility: Changing the Policies and Culture of Cell Line Authentication', Nat Methods, 12 (2015), 493-7.

[6] W. Parson, R. Kirchebner, R. Muhlmann, K. Renner, A. Kofler, S. Schmidt, and R. Kofler, 'Cancer Cell Line Identification by Short Tandem Repeat Profiling: Power and Limitations', FASEB J, 19 (2005), 434-6.
