# Supplementary material for: Benzoxazole Derivative K313 Induces Cell Cycle Arrest, Apoptosis and Autophagy Blockage and Suppresses mTOR/p70S6K Pathway in Nalm-6 and Daudi Cells
Source: Molecules. 2020 Feb 21;25(4):971. doi: 10.3390/molecules25040971 (PMC7070478; doi:10.3390/molecules25040971)
Supplement: Supplementary file 1 [file molecules-25-00971-s001.zip › Supplementary Materials/STR Authentication NALM-6.pdf]

Report of Human Cell Line Authentication

Analysis Date: 2017/04/10

Methods and Procedures

1. DNA is amplified with STR Multi-amplification Kit (Fluorescence Detection Kit);
2. PCR products are assayed with DNA Analyzer (Applied Biosystems®);
3. Appropriate positive and negative controls were run and confirmed for each sample submitted.
4. Comparision of the sample STR loci with an International Database is shown.

Results

1. The STR profiles of the sample are shown in Figure 1.
2. The comparision information is shown in Figure 2.
3. Conclusions are made according to the articles shown below:

   - A Resource for Cell Line Authentication, Annotation and Quality Control, **Nature**, 520(2015), 307-11.
   - Authentication of Human Cell Lines by Str DNA Profiling Analysis', (2013).

Conclusions

① **Obvious contamination of other human cell line is not found.**

② **According to the comparison information, the sample absolutely matches with a human cell line which is named as "Nalm-6".**

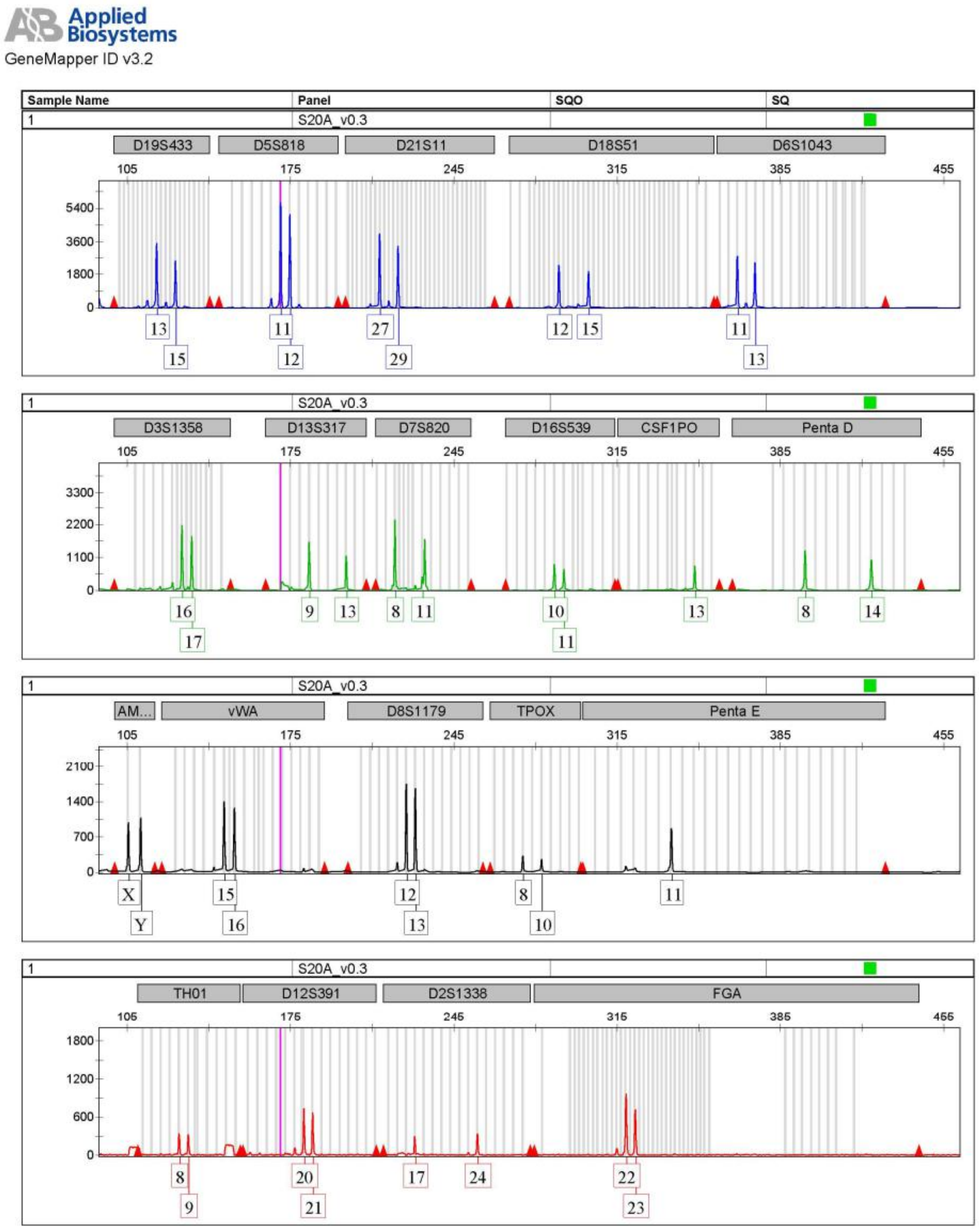

Figure 1. **STR profiles of the sample**

Result of STR matching analysis by your data.

- DSMZ Profile Database -

A graphical presentation is shown at the bottom of this page.

EV	Cell No.	Cell name	Locus names									Figures
			D5S818	D13S317	D7S820	D16S539	VWA	TH01	AM	TPOX	CSF1PO	
	Query (Your Cell)		*11, 12*	*9, 13*	*8, 11*	*10, 11*	*15, 16*	*8, 9*	*x, y*	*8, 10*	*13, 13*	
1.00(36/36)	RCB1933	NALM-6	11, 12	9, 13	8, 11	10, 11	15, 16	8, 9	X, Y	8, 10	13, 13	-

Figure 2. Comparision of the sample STR loci with an International Database

References:

[1] M. Yu, S. K. Selvaraj, M. M. Liang-Chu, S. Aghajani, M. Busse, J. Yuan, G. Lee, F. Peale, C. Klijn, R. Bourgon, J. S. Kaminker, and R. M. Neve, 'A Resource for Cell Line Authentication, Annotation and Quality Control', **Nature**, 520 (2015), 307-11.

[2] Yvonne Reid, PhD,1 Douglas Storts, PhD,2 Terry Riss, PhD,3,* and Lisa Minor,PhD4,*Authentication of Human Cell Lines by Str DNA Profiling Analysis', (2013).

[3] John Butler, 'Short Tandem Repeat Typing Technologies Used in Human Identity Testing', BioTechniques, 43 (2007), Sii-Sv.

[4] Hao Fan, and Jia-You Chu, 'A Brief Review of Short Tandem Repeat Mutation', Genomics, Proteomics & Bioinformatics, 5 (2007), 7-14.

[5] L. P. Freedman, M. C. Gibson, S. P. Ethier, H. R. Soule, R. M. Neve, and Y. A. Reid, 'Reproducibility: Changing the Policies and Culture of Cell Line Authentication', Nat Methods, 12 (2015), 493-7.

[6] W. Parson, R. Kirchebner, R. Muhlmann, K. Renner, A. Kofler, S. Schmidt, and R. Kofler, 'Cancer Cell Line Identification by Short Tandem Repeat Profiling: Power and Limitations', FASEB J, 19 (2005), 434-6.

[7] R. Wooster, A. M. Cleton-Jansen, N. Collins, J. Mangion, R. S. Cornelis, C. S. Cooper, B. A. Gusterson, B. A. Ponder, A. von Deimling, O. D. Wiestler, and

et al., 'Instability of Short Tandem Repeats (Microsatellites) in Human Cancers', Nat Genet, 6 (1994), 152-6.

[8] Jaiprakash G. Shewale, 'Str Profiling of Human Cell Lines: Challenges and Possible Solutions to the Growing Problem', Journal of Forensic Research, s2 (2011).
