# Supplementary material for: Benzoxazole Derivative K313 Induces Cell Cycle Arrest, Apoptosis and Autophagy Blockage and Suppresses mTOR/p70S6K Pathway in Nalm-6 and Daudi Cells
Source: Molecules. 2020 Feb 21;25(4):971. doi: 10.3390/molecules25040971 (PMC7070478; doi:10.3390/molecules25040971)
Supplement: Supplementary file 1 [file molecules-25-00971-s001.zip › Supplementary Materials/Supplementary Materials.docx]

Article

K313 induces cell cycle arrest, apoptosis, autophagy blockage and suppresses mTOR/p70S6K pathway in Nalm-6 and Daudi cells

Wenying Zhong 1, Xinwen Tang 2, Yang Liu 2, Chunyu Zhou 3, Pan Liu 4, Enhui Li 3, Peilin Zhong 3, Haoxue Lv 3, Qiang Zou 2,* and Maolin Wang 1,*

^1^ Key Laboratory of Bio-resources and Eco-environment of the Ministry of Education, College of Life Sciences, Sichuan University, Chengdu, 610065, Sichuan, PR China; txwzwy@163.com (W.Z.); mlwang@scu.edu.cn (M.W.)

^2^ Center of Science and Research, Chengdu Medical College, Chengdu, Chengdu, 610513, Sichuan, China; tangxinwei11@gmail.com (X.T.); scunn519@gmail.com (Y.L.); qiangzou99@gmail.com (Q.Z.)

^3^ School of Pharmacy, Chengdu Medical College, Chengdu, 610083, Sichuan, China; chunyu532@foxmail.com(C.Z.); CoooCaaa@163.com (E.L.); zhongpeiling33@163.com (P.Z.); lv1137641610@163.com (H.L.);

^4^ College of Biological Science and Technology, Chengdu Medical College, Chengdu, 610500, Sichuan, China; liupan145@gmail.com

* Correspondence: mlwang@scu.edu.cn (M.W.); qiangzou99@gmail.com (Q.Z.); Tel.: +86-028-85418776 (M.W.); +86-028-62739159(Q.Z.)

**Supplemental Material**


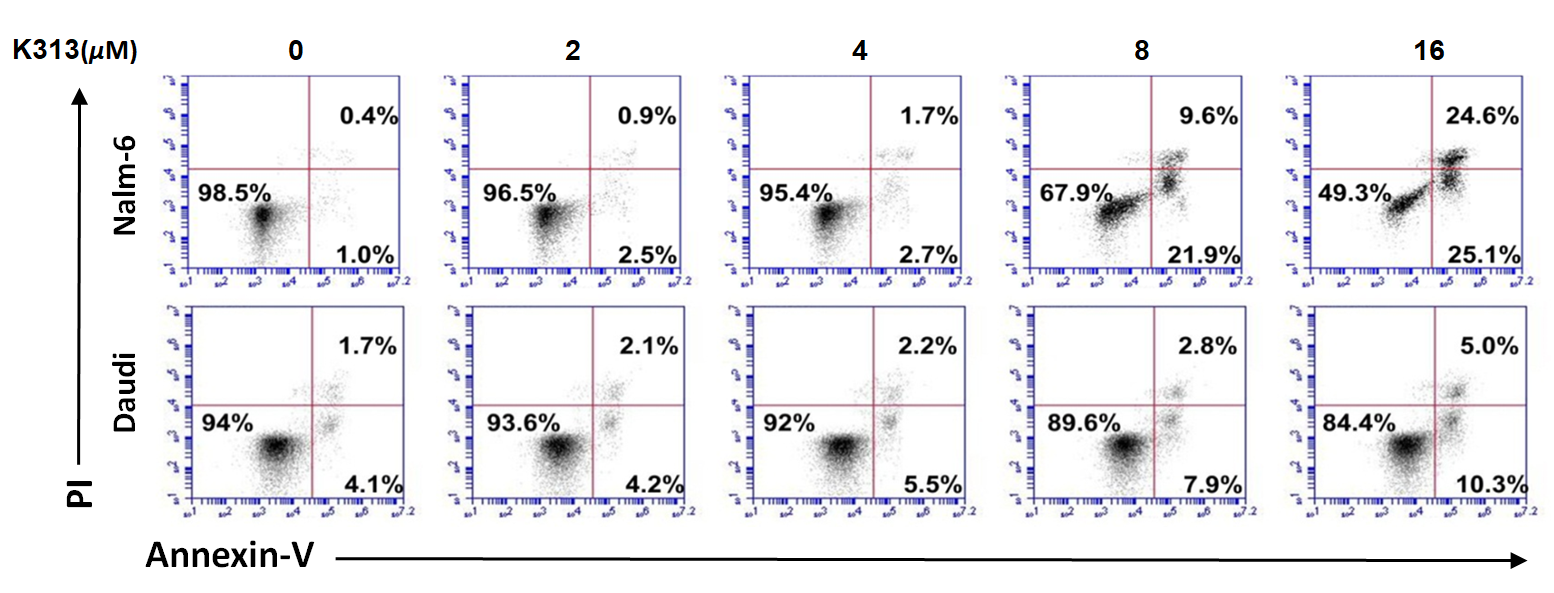


**Figure S1.** Detection of apoptosis in Nalm-6 and Daudi cells after treated with K313 for 24 h. The bottom-right corner stands for the apoptotic population.


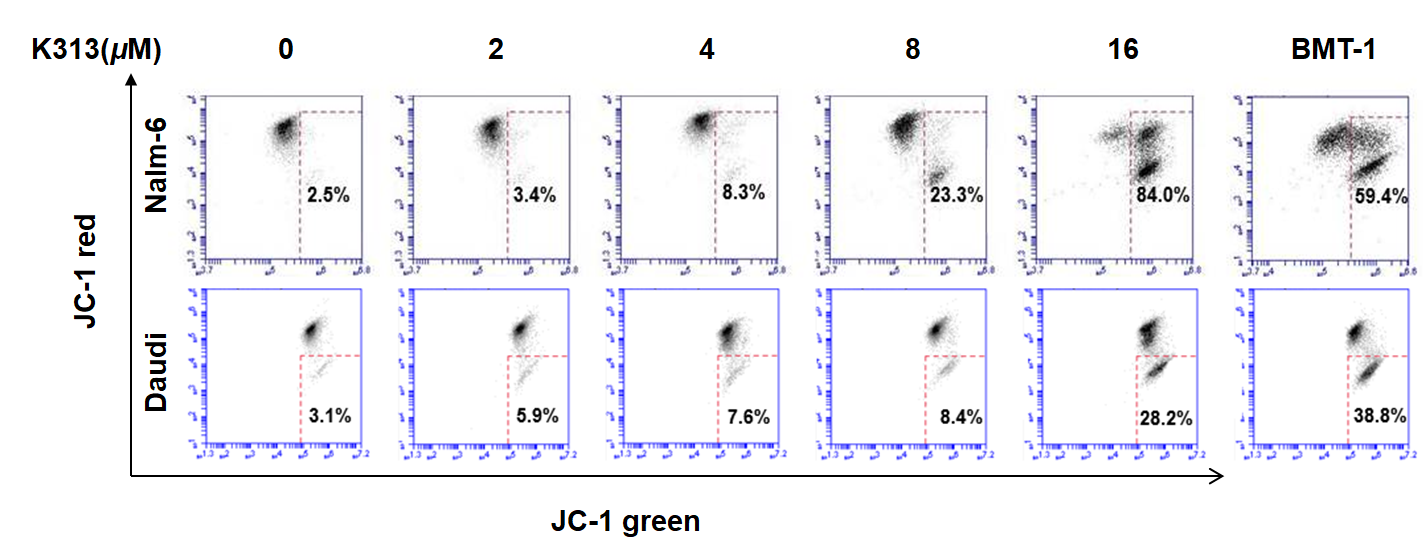


**Figure S2.** Detection of the loss of the mitochondrial membrane potential in Nalm-6 and Daudi cells after treated with K313 for 24 h. 20μM BMT-1 was used as positive control as confirmed repeatedly in our lab.


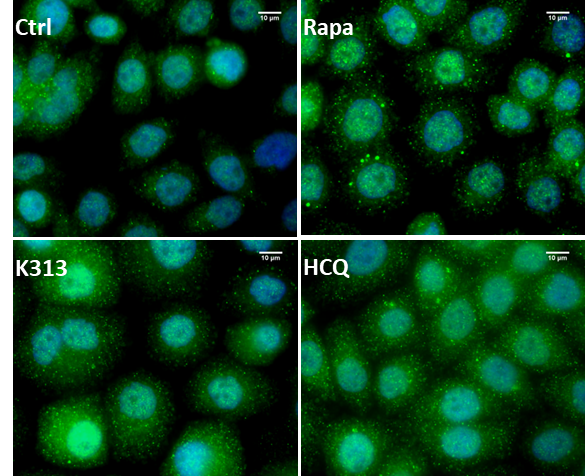


**Figure S3.** Observation of LC3 fluorescent puncta in hela cells under different treatments. Hela cells were treated with DMSO(Control), 5μM rapamycin, 16μM K313 and 20μM HCQ for 24 h. Then the cells of each group were fixed with 4% paraformaldehyde and stained with LC3 first antibody and Alexa Fluor 488-conjugated second antibody. LC3 fluorescent puncta were observed by fluorescence microscope.
